# Germline *RB1* Mutation in Retinoblastoma Patients: Detection Methods and Implication in Tumor Focality

**DOI:** 10.1167/tvst.11.9.30

**Published:** 2022-09-29

**Authors:** Duangnate Rojanaporn, Sermsiri Chitphuk, Nareenart Iemwimangsa, Takol Chareonsirisuthigul, Duangporn Saengwimol, Rangsima Aroonroch, Usanarat Anurathathapan, Suradej Hongeng, Rossukon Kaewkhaw

**Affiliations:** 1 Department of Ophthalmology, Faculty of Medicine Ramathibodi Hospital, Mahidol University, Bangkok, Thailand; 2 Research Center, Faculty of Medicine Ramathibodi Hospital, Mahidol University, Bangkok, Thailand; 3 Center for Medical Genomics, Faculty of Medicine Ramathibodi Hospital, Mahidol University, Bangkok, Thailand; 4 Department of Pathology, Faculty of Medicine Ramathibodi Hospital, Mahidol University, Bangkok, Thailand; 5 Department of Pediatrics, Faculty of Medicine Ramathibodi Hospital, Mahidol University, Bangkok, Thailand; 6 Program in Translational Medicine, Faculty of Medicine Ramathibodi Hospital, Mahidol University, Bangkok, Thailand; 7 Chakri Naruebodindra Medical Institute, Faculty of Medicine Ramathibodi Hospital, Mahidol University, Samut Prakan, Thailand

**Keywords:** retinoblastoma, germline mutation, *RB1*, hotspot exon, tumor focality

## Abstract

**Purpose:**

The study aimed to generate a stepwise method to reduce the workload of full-scale *RB1* sequencing for germline mutation screening in retinoblastoma (RB) patients. The implication of germline mutation in tumor focality was also determined in this study.

**Methods:**

A stepwise method was created on the basis of “hotspot” exons analyzed using data on germline *RB1* mutation in the *RB1*–Leiden Open Variation Database and then tested for mutation screening in the blood DNA of 42 patients with RB. The method was compared with the clinical next-generation sequencing (NGS) panel in terms of sequencing outcomes. The germline *RB1* mutation was examined in association with multifocality in RB.

**Results:**

Germline *RB1* mutation was identified in 61% of all bilateral cases in the first step of the 3 stepwise method and in 78% and 89% for the two and three steps combined, respectively. NGS detected a mosaic variant of *RB1* that was not detected by the first two steps and increased the sensitivity from 78% to 83%. Analysis of the relationship between mutation status and tumor focality indicated that multifocality in RB was dependent on germline *RB1* mutation, confirming a higher tendency to have a germline *RB1* mutation in patients with multifocal RB.

**Conclusions:**

A 3 stepwise method reduces the workload needed for sequencing of the *RB1* for bilateral cases. NGS outweighs conventional sequencing in terms of the identification of germline mosaic variants. Multifocal tumors in RB may be used to presume germline mutation.

**Translational Relevance:**

The presence of “hotspot” exons of germline *RB1* mutation in bilateral cases facilitates a mutation screening. However, when genetic testing is not available, multifocality in RB regardless of tumor laterality is predictive of germline *RB1* mutation.

## Introduction

Retinoblastoma (RB) is a childhood cancer of the retina initiated upon biallelic inactivation of the *RB1* tumor suppressor gene. The germline *RB1* mutations have been reported in 35% to 45% of all RB cases^1^ and can be passed to future offspring and put affected individuals at an increased lifelong risk of developing subsequent malignant neoplasms.^2^^,^^3^ Although clinical genetic testing is not available, patients with bilateral, multifocal, and/or familial presentation are classified into the heritable RB group, whereas sporadic cases with unilateral RB comprise the non-heritable RB group.^4^ However, heritable RB has been reported in 10% to 15% of non-familial unilateral RB cases.^1^^,^^5^^,^^6^ Additionally, germline mosaicism for *RB1* mutation has been identified in sporadic cases and may be associated with a lower risk of transmitting the pathogenic variants to offspring.^5^^,^^7^ Genetic testing for germline *RB1* mutation is thus crucial for precise genetic counseling and clinical management of RB patients.

Multiple screening strategies have been reported to identify *RB1* mutations, and no single technology is fully sensitive and efficient at detecting all mutations. A combination of tests is thus necessary for the molecular diagnosis of *RB1* mutations.^8^^–^^11^ Sequencing-based techniques, including Sanger sequencing and next-generation sequencing (NGS), enable the identification of small pathogenic variants. Large *RB1* deletions or duplications at exonic or chromosomal levels can be detected by multiplex ligation-dependent probe amplification (MLPA), quantitative multiplex polymerase chain reaction, or array-based techniques (array comparative genomic hybridization and single-nucleotide polymorphism arrays). Because small-scale mutations are often found as germline *RB1* mutations, Sanger sequencing or NGS was first employed to identify pathogenic variants, followed by MLPA or other techniques for detection of large-scale mutations that typically cannot be detected by sequencing-based methods.^10^^,^^12^^–^^14^ Recently, NGS analysis has permitted the identification of *RB1* copy number variation.^11^^,^^15^^–^^18^ However, Sanger sequencing and MLPA have been typically used to validate the findings from NGS.^11^^,^^15^^–^^18^

Sanger sequencing has long been considered a gold-standard method, although sequencing a total of 27 exonic sequences with the flanking intronic regions and the promoter sequence can be laborious. Although mutations are dispersed in the *RB1* gene, previous studies have suggested that “hotspot” exons where germline *RB1* mutations are frequently detected may exist.^1^^,^^10^^,^^19^ The order of exons could accordingly be prioritized for analysis of pathogenic variants, which could reduce the overall workload.

To this end, we aimed to devise a stepwise method based on “hotspot” exons analyzed using data on germline *RB1* mutation in the *RB1*–Leiden Open Variation Database (rb1-lsdb, http://d-lohmann.de/variants.php?action=search_all; see Supplementary Material for the retrieved data). The stepwise method using Sanger sequencing combined with MLPA was then tested for *RB1* mutation screening in blood DNA of our patients. We also compared the stepwise method with the NGS-based method in terms of sequencing outcomes to demonstrate its advantages and disadvantages. Furthermore, we provide the evidence for the relationship between germline mutation and multifocality in RB, which is useful for the prediction of germline *RB1* mutation when genetic testing is not available.

## Methods

### Blood Samples

Blood was collected on diagnosis or during a follow-up visit from 42 patients with RB who presented at Ramathibodi Hospital between 2018 and 2021 for genetic testing. The characteristics of the patients with RB are provided in Table 1. All experimental protocols were approved by the Human Research Ethics Committee, Faculty of Medicine Ramathibodi Hospital, Mahidol University (protocol number ID07-60-14) and performed in accordance with the tenets of the Declaration of Helsinki. Before the samples were collected, informed consent was obtained from each patient's parent.

**Table 1.** Characteristics of Retinoblastoma Patients

	Laterality of Tumors		
	Bilateral	Unilateral	Total
Patients, *n* (%)	18 (43)	24 (57)	42 (100)
Sex, *n*			
Male	9	15	24
Female	9	9	18
Age of diagnosis (mo), mean ± SD	11.5 ± 8.4	17 ± 14.1	—
ICRB (all eyes), *n*			
A	1	0	1
B	5	1	6
C	5	0	5
D	4	11	15
E	15	10	25
Extraocular	1	2	3
IIRC (all eyes), *n*			
A	1	0	1
B	5	1	6
C	5	1	6
D	9	14	23
E	9	5	14

Data for five and 10 eyes are not available for grouping based on the Intraocular Classification of Retinoblastoma (ICRB) and International Intraocular Retinoblastoma Classification (IIRC), respectively.

### DNA Extraction

Genomic DNA was extracted with the DNeasy Blood & Tissue Kit (Qiagen, Hilden, Germany) from peripheral blood mononuclear cells isolated from blood with Ficoll-Paque Plus reagent (GE Healthcare, Uppsala, Sweden). The DNA quality and quantity were examined by agarose gel electrophoresis and a NanoDrop 2000 spectrophotometer (Thermo Fisher Scientific, Waltham, MA), respectively.

### Analysis of “Hotspot” Exons

Data on germline *RB1* mutations identified in unilateral and bilateral RB/retinoma cases were retrieved from the *RB1*-Leiden Open Variation Database for the analysis. The *RB1*-Leiden Open Variation Database is the free-access database in which *RB1* mutations, including the germline *RB1* mutations in retinoblastoma/retinoma patients, have been deposited. The genotypes and phenotypes of patients and tissue type in which the variant was detected are reported. A detailed description of data extraction is provided in the Supplementary Method and Supplementary Figure S1A. The association between exons with reported mutations and tumor laterality was analyzed using the χ^2^ test. The order of exons was prioritized based on “hotspot” exons, which had more frequently identified mutations than the median number of mutations per exon.

### Sanger Sequencing, MLPA, and Data Analysis of *RB1* Variants

PCR conditions for *RB1* gene amplification including the promoter and exons 1 to 25 with flanking intronic sequences, Sanger sequencing, and MLPA were as previously described.^10^ PCR reaction was performed by using Applied Biosystems AmpliTaq Gold 360 master mix (Thermo Fisher Scientific). All primer sequences are provided in the Supplementary Table S1. MLPA was conducted using the SALSA MLPA P047-D1 *RB1* probe mix (MRC-Holland, Amsterdam, The Netherlands). *RB1* variants were analyzed, and the pathogenicity of variants was predicted using an online bioinformatics tool as described previously.^10^ A condition of allele-specific PCR is described in the Supplementary Method.

### Exon-Capture Sequencing by NovoFocus CR Clinical NGS Panel

Targeted NGS was performed on blood DNA from 42 patients with RB using a NovoFocus CR clinical panel (Novogene, Beijing, China) targeting exons of 106 genes associated with hereditary cancers, as well as a NovaSeq Sequencer (the read length was 150 bp and the average coverage depth of the *RB1* gene was 500×).

### NGS Analysis

The raw sequencing data (FastQ) were generated, assembled, and aligned against the reference gene sequence based on human genome build GRCh38/hg38. All data processing and variant calling were conducted using bwa-0.7.17, gatk-4.1.6.0, MarkDuplicates, Samtools, BQSR, and HaplotypeCaller from the Best Practices workflows developed by the Genomic Analysis ToolKit (GATK) team at the Broad Institute (Cambridge, MA). All variants were first filtered by a minimum of 10× coverage and a Phred quality score greater than Q20. Variants were annotated, and the effect of variants was predicted using VarSeq 2.2.1 (https://www.goldenhelix.com/products/VarSeq/; Golden Helix, Inc., Bozeman, MT). Briefly, variants with a population allele frequency <1% in any population from 1000 Genome Phase 3 and gnomAD were selected. The impact of variants was predicted using Mutation Taster, Mutation Assessor, metaLR, FATHMM, SIFT, PolyPhen2, and dbscSNV. Selected variants were also classified for pathogenicity using the standards and guidelines for the interpretation of sequence variants recommended by the American College of Medical Genetics and Genomics and the Association for Molecular Pathology.^20^

### Analysis of Tumor Focality

All patients underwent an examination under anesthesia. Under anesthesia, each patient received a comprehensive eye examination using binocular indirect ophthalmoscopy with 360° indentation, Schiotz tonometer, and B-scan ultrasonography. Tumor focality was examined and documented by fundus imaging at the first visit using a wide-angle contact fundus camera (RetCam 3, Clarity Medical Systems, Inc., Pleasanton, CA). Primary retinal tumors were differentiated from the intraocular seeding compartments according to the previous report.^21^ Data on tumor focality were obtained from the patients in this study and a group of patients in our previous study of germline *RB1* mutation^10^ (Supplementary Table S2). The relationship between tumor focality (unifocal and multifocal presentation) and the mutation status (germline and non-germline *RB1* mutation) was examined using Pearson's χ^2^ test. The difference in mean age at diagnosis was determined between unifocal and multifocal presentation using the unpaired two-samples Wilcoxon test. The statistical significance was measured at *P* < 0.05.

## Results

### A Stepwise Method for Detection of a Germline *RB1* Mutation

**Figure 1.**
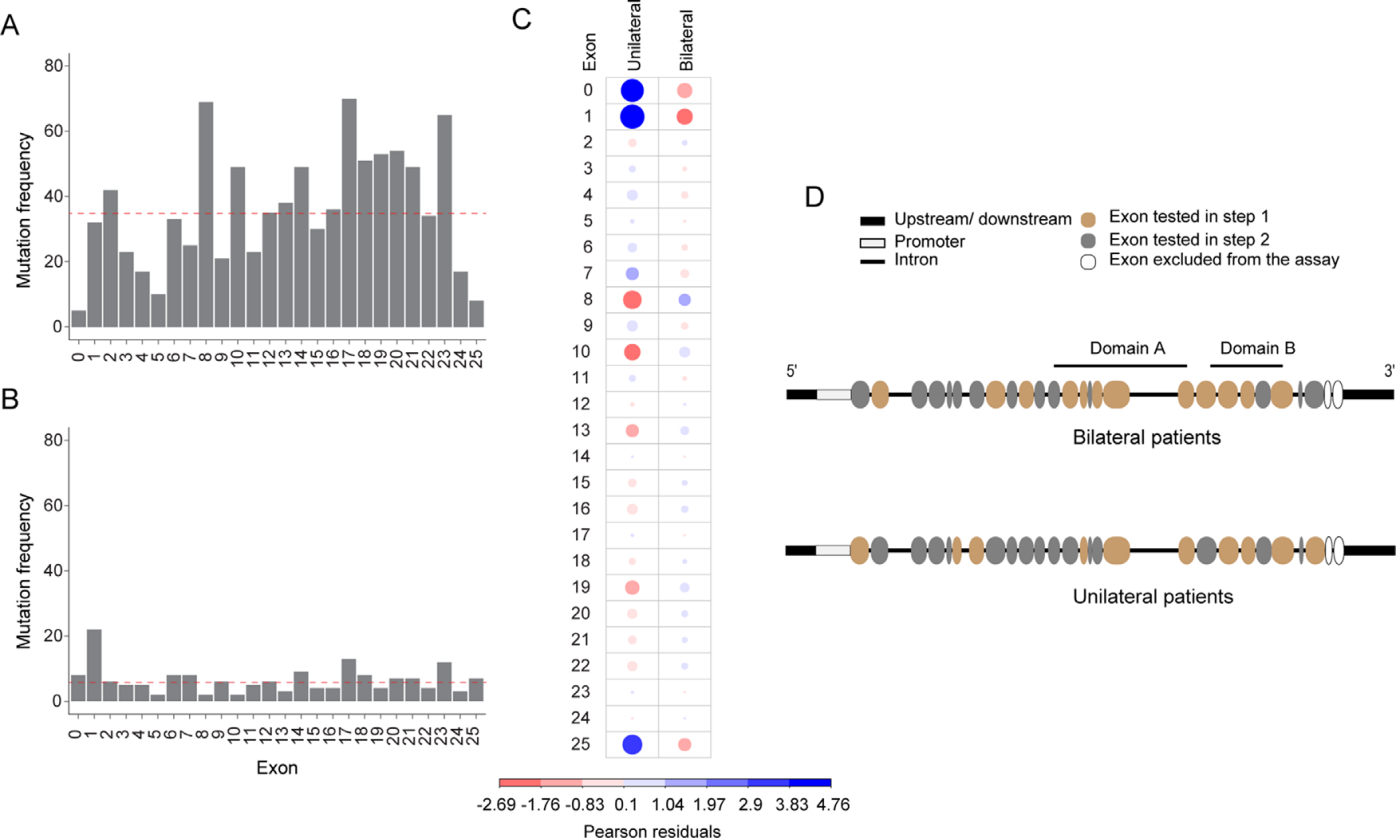
A stepwise method for detection of germline *RB1* mutations in patients with RB. (**A**, **B**) Frequency of mutations in promoter and exons/introns of the *RB1* gene in patients with germline mutations from rb1-lsdb; *n* = 938 mutations for patients with bilateral RB (**A**) and *n* = 170 mutations for patients with unilateral RB (**B**). The *red broken lines* in (**A**) and (**B**) indicate the median. (**C**) Plot of Pearson residuals extracted from Pearson's χ^2^ test. Positive residuals (*blue*) and negative residuals (*red*) specify positive and negative associations between the regions and tumor laterality; 0 indicates the promoter. See Supplementary Figure S1B for the percent relative contribution of each cell in the contingency table to the total χ^2^ score. (**D)** Schematic illustrating the *RB1* gene in which exons are first and second prioritized in a stepwise method for patients with bilateral (*top*) and unilateral (*bottom*) RB.

Germline *RB1* mutations in patients with unilateral and bilateral RB were extracted from the rb1-lsdb database (Supplementary Material; Supplementary Fig. S1A; Figs. 1A, 1B). We found that exons/introns with reported mutations were significantly associated with RB laterality (*P* = 1.075 × 10^−8^) (Fig. 1C). Exons 8 and 10 were strongly associated with patients with bilateral RB, whereas exons 1 and 25 and the promoter were strongly associated with the patients with unilateral RB (Supplementary Fig. 1B, Fig. 1C). We then generated two independent stepwise methods for patients with bilateral and unilateral RB in detecting germline *RB1* mutation. Of the 42 patients, 18 patients (43%) were affected bilaterally, and 24 patients (57%) were affected unilaterally; the clinical demographics are shown in Table 1.

For patients with bilateral RB, the median frequency of mutations in exons/introns was 35. The 12 regions that had a mutation frequency greater than 35 were considered hotspots and thus were prioritized for mutation screening (exons 17, 8, 23, 20, 19, 18, 10, 14, 21, 2, 13, and 16) (Figs. 1A, 1D). This step was followed by mutation screening in the other 14 regions with a mutation frequency ≤ 35 (exons 12, 22, 6, 1, 15, 7, 3, 11, 9, 4, 24, 5, 25, and the promoter) (Fig. 1D). MLPA was then used to detect large-scale deletion/duplication if pathogenic variants were not identified by the previous steps. Exons 26 and 27 were excluded from the assay, because mutations are reported very rarely in these exons. Mutations were identified in 61% (11/18) of patients with bilateral RB in the first step. Pathogenic variants were additionally identified in 17% (3/18) of the patients in step 2, resulting in a total of 78% for the two steps combined. Finally, MLPA detected a large-scale deletion in the *RB1* gene in 11% (2/18) of patients. However, mutations in 11% of patients (2/18; 553N100 and 999N107) remained undetectable. Altogether, a detection rate of 89% (16/18) was reported in patients with bilateral RB using the stepwise method (Table 2). Likewise, the detection rate was consistent when this stepwise procedure was used with our previous group of patients,^10^ making the detection rates 58% ± 5%, 77 % ± 2%, and 91% ± 2% (mean ± SD from two studies) for the first step alone, first two steps combined, and all steps, respectively. Additionally, classification of data previously reported by others into steps 1 and 2 suggests that the detection rate was consistent with the current study, except the studies from Vietnam and one group from India (36% and 33% for step 1) (Table 3). This finding indicated that “hotspot” exons are present in the *RB1* gene, with more than 50% of germline mutations located in the exons grouped in step 1 (Table 3).

**Table 2.** Summary of Germline *RB1* Mutations Identified in Retinoblastoma Patients by NGS and a Stepwise Method

ID	Phenotype	g-Position (L11910.1)	cDNA Change (NM_000321.3)	Ex/In	Expected Consequence	Times Reported in rb1-lsdb (*n*)
026 N72	Bi	g.70330G>A	c.1215+1G>A	In12	Splice	64
183 N88	Bi	g.150113 A>G	c.1811A>G	Ex18	p.Asp604Gly	3
733 N92	Bi	g.76460C>T	c.1363C>T	Ex14	p.Arg455X	62
321 N94	Bi	g.162366delA	c.2488delA	Ex23	p.Arg830Glufs*3	Novel
553 N100^a^	Bi	g.78238C>T	c.1654C>T	Ex17	p.Arg552X	70
184 N108	Bi	g.150119A>C	c.1814+3A>T	In18	Splice	1
921 N115	Bi	g.76898C>T	c.1399C>T	Ex15	p.Arg467X	55
133 N118	Bi	g.78198dupA	c.1614dupA	Ex17	p.Glu539Argfs*16	Novel
105 N119	Bi	g.76460C>T	c.1363C>T	Ex14	p.Arg455X	62
509 N122	Bi	g.45798G>A	c.540-1G>A	In5	Splice	6
930 N123	Bi	g.5553A>C	c.264+3A>C	In2	Splice	1
863 N124	Bi	g.59728C>T	c.796C>T	Ex8	p.Gln266X	3
196 N138	Bi	g.162369T>G	c.2489+2T>G	In23	Splice	Novel
138 N144	Bi	g.150037C>T	c.1735C>T	Ex18	p.Arg579X	94
496 N147	Bi	g.77046C>A	c.1467C>A	Ex16	p.Cys489X	4
138 N126	Uni	g.150037C>T	c.1735C>T	Ex18	p.Arg579X	94
417 N146	Bi	Whole gene deletion				
373 N153	Bi	Whole gene deletion				

Variants are classified for pathogenicity based on the variant classification of the American College of Medical Genetics and Genomics Standards and Guidelines.^20^ Bi, bilateral; Uni, unilateral; Ex, exon; In, intron.

a Pathogenic variant was detected by NGS.

**Table 3.** Detection Rate of *RB1* Germline Mutations by NGS Grouped Into Steps 1 and 2

			Detection Rate		Detection Rate by the	
			by NGS (%)		Stepwise Method^b^ (%)	
		Patients with	SNPs and InDels			
Reference	Country	Bilateral RB (*n*)	(Mosaicism)^a^	CNV	Step 1	Step 2
Zou et al.^18^	China	62	75 (2)	13	45	31
Hoang et al.^16^	Vietnam	25	80	20	36	44
Yousef et al.^27^ ^c^	Jordan	22	100	—	68	32
Singh et al.^11^	India	22	82	18	55	27
Li et al.^17^	USA	19	79	21	53	26
Amitrano et al.^26^	Italy	11	82 (9)	—	82	0
Devarajan et al.^15^	India	21	66	14	33	33
Grotta et al.^8^	Italy	29	76	—	48	28
Mean ± SD (%)	—	—	82 ± 9	17 ± 4	53 ± 16	28 ± 13

SNPs, single-nucleotide polymorphisms; InDels, insertions/deletions; CNV, copy number variation.

a Detection rate of low-level mosaic mutations that cannot be detected by Sanger sequencing.

b Excluding reported mosaic variants that cannot be detected by Sanger sequencing.

c Patients with bilateral RB include probands only.

For patients with unilateral RB, the median frequency of mutations in exons/introns was 6 (Fig. 1B). Eleven regions with a mutation frequency >6 were included in the first step (exons 1, 17, 23, 14, promoter, exons 6, 7, 18, 20, 21, and 25), followed by screening in the other 15 regions (exons 2, 9, 12, 3, 4, 11, 15, 16, 19, 22, 13, 24, 5, 8, and 10), and then MLPA if no variants were detected (Figs. 1B, 1D). A mutation was identified in only one patient (138N126) from 24 patients with unilateral RB by step 1 (Table 2). The results suggest that stepwise detection of germline *RB1* mutations based on “hotspot” exons/introns was feasible for patients with bilateral RB.

### Targeted NGS of the *RB1* Gene

**Figure 2.**
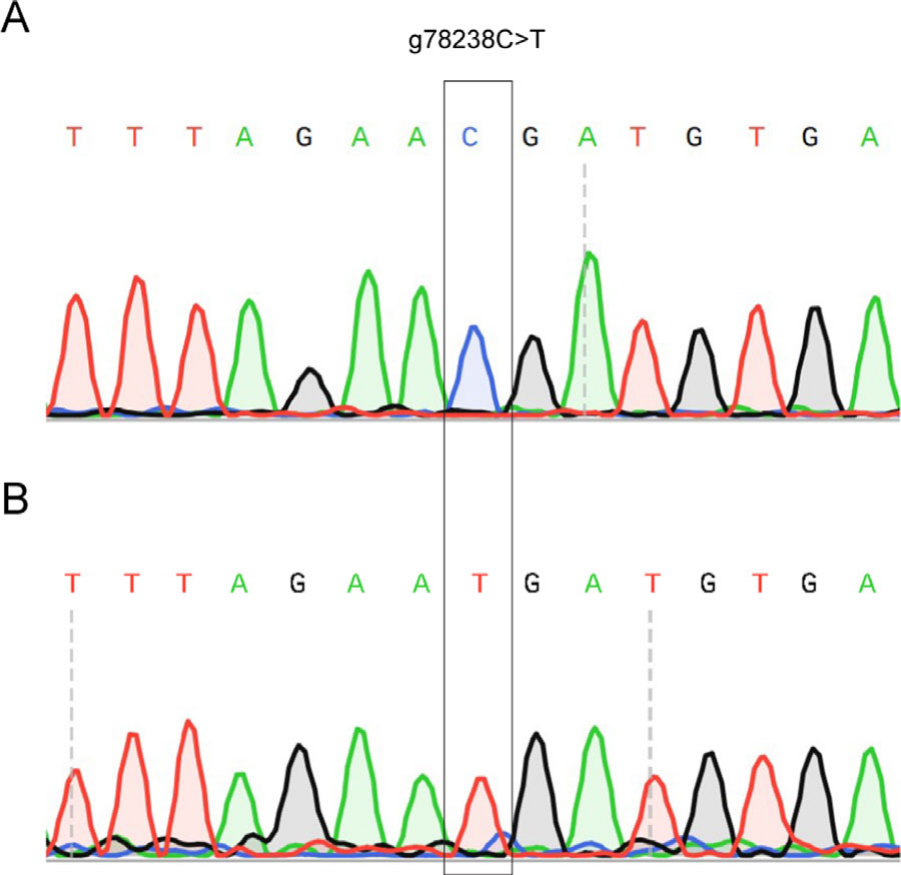
A mosaic pathogenic variant is detected by allele-specific PCR followed by Sanger sequencing. (**A**, **B**) Chromatograms indicate detections of wild-type allele by normal PCR condition (**A**) and of mosaic germline mutation by allele-specific PCR (**B**) in sample 553N100.

NGS was performed on the same blood DNA samples of patients to compare the sensitivity and accuracy with the stepwise method. The NovoFocus CR clinical NGS panel was used to evaluate germline alterations in 106 genes associated with hereditary cancers including *RB1*. We found that NGS detected pathogenic variants in 83% (15/18) of patients with bilateral RB (Table 2). This detection rate was higher than that using the first two steps (78%, 14/18) of the stepwise method. A mutation in exon 17 (g.78238C>T), which was not detected by the stepwise method, was identified by NGS for sample 553N100 (Table 2). This suggests that a mosaic variant was present in sample 553N100 for which NGS enabled the detection. The mosaic pathogenic variant was confirmed by allele-specific PCR, followed by Sanger sequencing (Fig. 2). However, one sample (999N107) remained undetectable by NGS, consistent with Sanger sequencing. The detection rate for small-scale mutations (single-nucleotide polymorphisms and insertions/deletions) by NGS was consistent with other reports (Table 3). In addition, the detection rate was 94% (17/18) when MLPA was used in conjunction with NGS, which was higher than the stepwise detection for patients with bilateral RB (89%).

Like Sanger sequencing, NGS detected a mutation in one unilateral RB patient (138N126) from a total of 24 patients (Table 2). No mutations were detected by MLPA for the unilateral patients. Altogether, patients carrying a germline *RB1* mutation accounted for 43% of all cases. Three variants in unrelated patients were novel and associated with disease-causing mutations by in silico pathogenicity analysis (Supplementary Table S3). Nonsense mutations (44%) were the most frequently detected mutations, followed by splice (27.5%), frameshift (11%), gross deletion (11%), and missense (5.5%) mutations (Table 2).

Our results suggest that the stepwise method is applicable for detecting a germline *RB1* mutation in patients with bilateral RB. In contrast, the small proportion of patients with unilateral RB with germline mutations (4% of all cases) made the stepwise method inefficient; thus, NGS became the more appropriate approach for mutation screening.

In addition to germline *RB1* mutations, we identified *BARD1* variants in 12.5% (2/16) of patients carrying heritable RB. Two *BARD1* missense variants, c.1601C>T; p. Thr534Ile and c.2191C>T; p. Arg731Cys, simultaneously existing in *cis*, were detected and identical in two unrelated patients with germline *RB1* mutation (733N92 and 321N94). The variants found in *cis* might cause dominant-negative effect, as previously described in other diseases,^22^ while double mutations in *cis* in *BARD1* are reported in a study of a family with hereditary breast and ovarian cancer.^23^

### Germline *RB1* Mutation and Tumor Focality in Retinoblastoma

**Figure 3.**
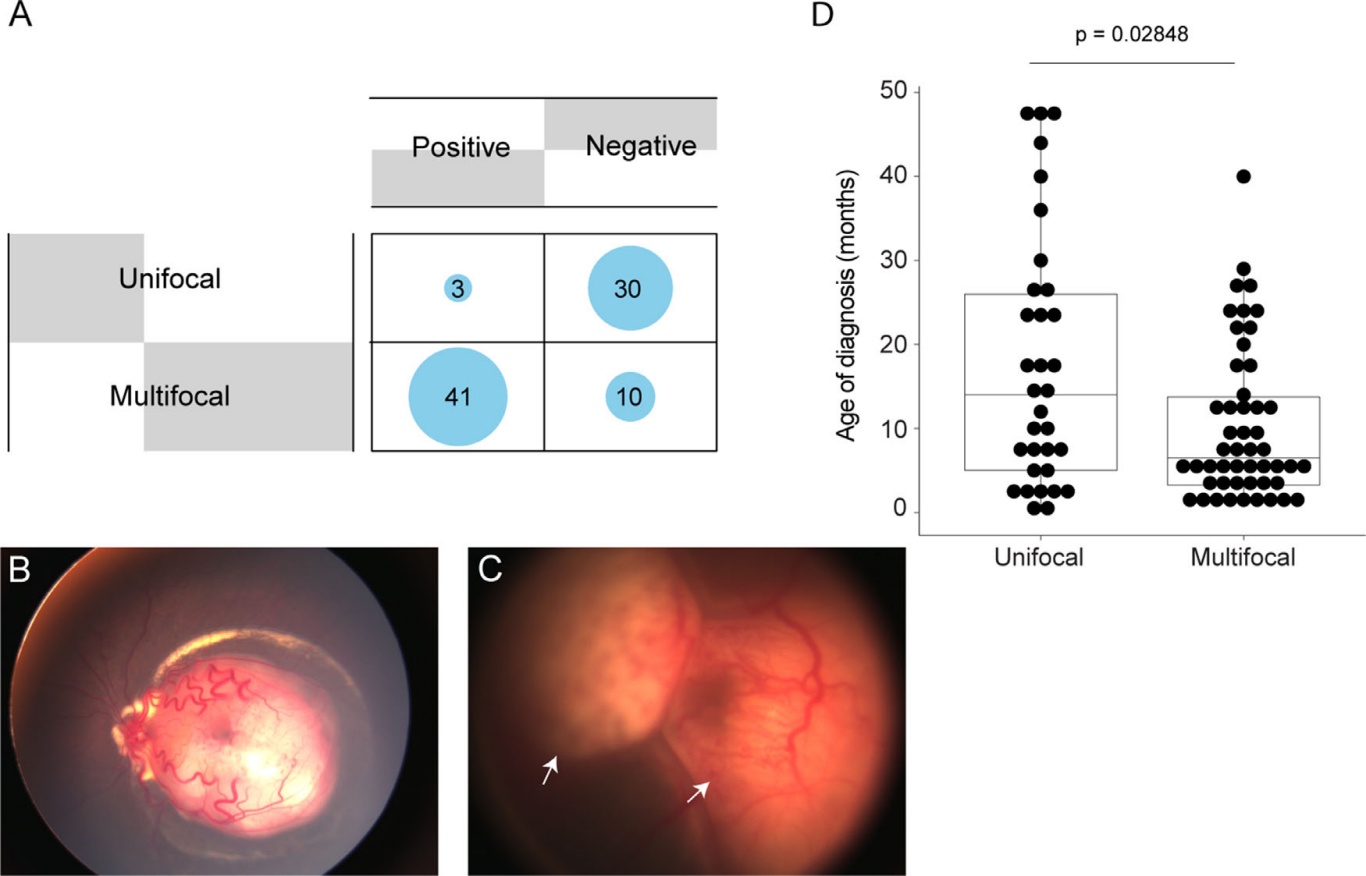
Relation of the tumor focality to mutation status and age of diagnosis. (**A**) Balloon plot showing the mutation status and tumor focality. (**B**, **C**) Fundus photographs show unifocal RB of the patient (age of diagnosis was 8 months) with germline mutation (**B**) and multifocal RB (arrow) of the patient (age of diagnosis was 2 months) with undetectable germline mutation (**C**). (**D)** The range of age of diagnosis (AOD) of RB patients with unifocal or multifocal tumors. Data were analyzed from 84 patients for (**A**), and 83 patients (due to the AOD of one patient with multifocal tumors not being available) for (**D**). Pearson's χ^2^ test was used to examined the relation of tumor focality to a germline mutation (*P* < 0.0001). The unpaired two-samples Wilcoxon test was used to test the difference in AOD. A statistical significance was *P* < 0.05.

We asked whether the presentation of tumor focality in RB diagnosed at the first visit is related to the status of germline *RB1* mutation. Germline mutation was examined in association with multifocality in RB using the data from the current and our previous^10^ studies (Supplementary Table S2). As expected, patients with unifocal RB (36%, 30/84) tested negative for germline *RB1* mutation, and 49% (41/84) of patients with multifocal tumors tested positive for germline mutations (Fig. 3A). However, we found that 4% (3/84) of patients with unifocal RB had germline mutations, and 12% (10/84) of patients with multifocal RB tested negative (Figs. 3A–3C). Statistically, tumor focality diagnosed at the first visit was significantly dependent on the status of germline *RB1* mutation based on mutation detection using Sanger sequencing and MLPA (*P* < 0.0001, χ^2^ test) (Fig. 3A); multifocality was associated with germline *RB1* mutation. The mean age at diagnosis of patients with unifocal tumors was later than the patients with multifocal tumors (17.8 and 10.3 months, respectively) (Fig. 3D). The result suggests that multifocal tumors could be used to presume germline *RB1* mutation in RB patients.

## Discussion

Access to genetic testing for germline *RB1* mutation is crucial for precise genetic counseling and clinical management of RB patients. We found that “hotspot” exons may exist for germline *RB1* mutation in patients with bilateral RB and could be used to generate a stepwise method where the first step detected a mutation in more than 60% of all patients. Consistently, germline pathogenic variants were often found in exons 8, 17, 14, 20, 23, and 10 in a large cohort study of 1404 patients^1^; these exons are included in the first step of detection for patients with bilateral RB. Of note, mutations in exons 8 and 23, encoding the linkers connecting structured regions of the RB protein, are strongly associated with the development of bilateral RB and early patient age and are thus considered “hotspots” for patients with bilateral RB.^24^^,^^25^ Additionally, a mutation in exon 18, encoding the linker connecting pockets A and B, is also often found in heritable RB^24^ and was found in our cohort. The exons encoding the A/B structured domain and linkers of the RB protein represent a “hotspot” where a germline mutation is frequently identified. Therefore, prioritizing genetic testing based on these “hotspot” exons can reduce the workload of full-scale *RB1* sequencing for mutation screening in patients with bilateral RB, as shown in this study.

The mutation rate of exons in the first and second steps is generally consistent with previous reports from different countries (Table 3). However, the mutation rates of exons reported from Vietnam and a group in India are distinct.^15^^,^^16^ Notably, the age of diagnosis is late from these two studies (Vietnam, 1–48 months, mean = 19 months; India, 0–66 months, mean = 20 months), and the detection rates of step 1 are lower than step 2.^15^^,^^16^ In contrast, the mean age of diagnosis was 10 to 12 months for the patients in our study and others, and the detection rate was high using the first step (Table 3).^18^^,^^26^^,^^27^ The later ages of diagnosis in India and Vietnam might relate to delayed presentation and high detection rate of step 2.

To prioritize the order of exons, our 3 stepwise method was derived based on the mutation frequency in exons, whereas the 4 stepwise method of previous work^28^ was created based on the frequency of nonsense variants in exons reported in rb1-lsdb. For patients with bilateral RB, our detection rate was relatively higher for each step of the 3 stepwise method (61%, 17%, and 11%) compared with the 4 stepwise method (46%, 15%, 8%, and 0%); our overall detection rate was 89% versus 69% in previous work using Sanger sequencing and MLPA.^28^ Interestingly, the detection rate of 61% by sequencing of 12 regions in the step 1 almost achieves the reported detection rate of full-scale *RB1* sequencing (71%–76%)^10^^,^^12^^–^^14^ in patients with bilateral RB. However, we found that the stepwise method is inefficient for detecting germline mutations in patients with unilateral RB because of the small proportion of unilateral patients carrying germline mutations (4%). Moreover, the number of patients with unilateral RB with germline mutations varies; for example, 33% and 4% of all cases were reported in our previous^10^ and current cohorts, respectively. This suggests that NGS is appropriate and efficient for mutation screening in patients with unilateral RB.

We and others found that the main advantage of NGS-based strategies for screening *RB1* mutation is the ability to detect mosaic variants that cannot be detected by Sanger sequencing.^5^^,^^18^^,^^26^ Germline mosaicism for g78238C>T (R552X) was detected in one of our patients with bilateral RB (553N100) at the sequencing depth of 490×, whereas an average sequencing depth of *RB1* region for all samples was 500×; this mosaic variant has been reported to be associated with maternal transmission causing RB in offspring.^29^ However, a mutation remained undetectable at the sequencing depth of 370× in the other patient with bilateral RB (999N107). This suggests that at least 500× or deeper sequencing^5^^,^^7^^,^^26^ may have been required to identify mosaic variants in this study, although the sequencing depth was out of our control due to prior design of the NGS panel available at the time of study. Additionally, the NGS panel used contains known 106 genes that are associated with hereditary cancers. The sequencing results from this NGS panel may provide us with the knowledge about other RB-relating genes in addition to *RB1*, which is a known benefit of using NGS, although identification of other variants is not the primary aim. We found that double mutations in *cis* in *BARD1* might relate to RB development.

We found that 4% of our patients with unifocal RB tested positive for germline *RB1* mutation and had no family history. It is feasible that these patients received a germline mosaic pathogenic variant from an asymptomatic parent, as previously described in patients with unilateral and unifocal RB.^30^ Additionally, these patients could have high-level germline mosaic variants, which are typically detected by Sanger sequencing.^5^^,^^7^ In contrast, 12% of patients with multifocal RB tested negative, which contradicts the association of multifocal tumors with germline mutation/heritable RB. The undetectable mutations in these patients could be due to insensitivity of the stepwise method to detect germline mosaic variants. However, statistically, we found that the multifocality in RB is related to germline mutation and may be used to predict the germline mutation for RB patients when genetic testing is not available.

The limitation of this study includes the small sample size; a larger sample size could enhance the confidence of the detection rate of each step of the 3 stepwise method. Identifying the germline status (either true or mosaic germline mutation) and relating it to tumor focality in a larger cohort of RB patients may be important to verifying this conclusion.

Altogether, our study is of use in prioritizing genetic testing where full NGS is not feasible. The 3 stepwise method reduces the workload of full-scale *RB1* sequencing for mutation screening in patients with bilateral RB. When available, NGS is efficient for mutation screening, especially in unilateral RB cases, and is able to identify mosaic variants. However, when genetic testing is not available, the multifocality in RB is predictive of germline *RB1* mutation.

## Supplementary Material

Supplement 1

Supplement 2
