# Supplementary material for: Germline RB1 Mutation in Retinoblastoma Patients: Detection Methods and Implication in Tumor Focality
Source: Transl Vis Sci Technol. 2022 Sep 29;11(9):30. doi: 10.1167/tvst.11.9.30 (PMC9527333; doi:10.1167/tvst.11.9.30)
Supplement: Supplement 2 [file tvst-11-9-30_s002.pdf]

Germline *RB1* mutation in retinoblastoma patients: detection methods and implication in tumor focality

Supplementary Method

Allele-specific PCR

The PCR reaction was performed in a total volume of 25 μl containing 50 ng genomic DNA, 0.1 μM each of forward and reverse LS primer, 0.5 μM of forward and reverse NLS primer, and 1X AmpliTaq Gold 360 master mix (Applied Biosystems, Foster City, CA). Following an initial denaturation step at 95°C for 10 min, product amplification was performed for a total of 35 cycles with the profile: 15 cycles of 95°C for 30 s, 58°C for 30 s, 72°C for 60 s to enrich the target locus harboring the SNP of interest (referred to as the first reaction phase). The second reaction phase consisted of 5 cycles of 95°C for 10 s and 45°C for 30 s, followed by 15 cycles each consisting of 95°C for 10 s, 53°C for 30 s and 72°C for 5 s. The final extension step was at 72 °C for 7 min, and reactions were held at 15 °C until collection. The reaction products were separated by electrophoresis on a 2% agarose gel and visualized by ethidium bromide staining. Products were purified using a gel extraction kit (Qiagen) for sequencing. The primer sequences included locus-specific primers (LS-F: 5′ gcatacactcaaaattggaaggctatttcctatgagtcc 3′ and LS-R: 5′ tcacacactggggcctgtcatg 3′) and nested locus-specific primers (NLS-F: 5′cccggaaatgataaaacatttagaac 3′ and NLS-R: 5′ cccttttgttttaaagtggc 3′).

Data on germline RB1 mutation

Germline *RB1* mutation data were obtained from the RB1-Leiden Open Variation Database. (rb1-lsdb, http://d-lohmann.de/variants.php?action=search_all). First, exons with reported mutations were selected based on germline in the column of Mut. Origin (origin of mutation) and unilateral and bilateral retinoblastoma/retinoblastoma/retinoma in the column of

Phenotype (disease phenotype of the patients). Second, the data were further filtered to ensure that the mutations were germline by selecting germline in the column of Originated_in (the cell of origin) or blood in the column of Tissue (tissue type in which the sequence variant was detected). Finally, the mutations were then selected based on unilateral or bilateral RB in the Phenotype column (Supplementary File and Supplementary Fig. S1A).

Supplementary Table S1. List of primers for PCR amplification and sequencing

Primer name	Primer ID	Primer sequence (5'-3')	Length (bp)	Size
Promoter_F	RbP1	CTGGACCCACGCCAGGTTTC	20	341
Promoter_R	RbP2	GTTTTGGGCGGCATGACGCCTT	22	
E1_F	RbP3	CCGGTTTTTCTCAGGGGACGTTG	23	342
E1_R	RbP4	TTGCGCCCGCCCTACGCACAC	21	
E2_F	RbP5	TGTTATGTGCAAACTATTGAAACAAG	26	359
E2_R	RbP6	AGGTAAATTTCCTCTGGGTAATG	23	
E3_F	RbP7	TGCCATCAGAAGGATGTGTTAC	22	411
E3_R	RbP8	TGGCAGTTCACTATTTGGTCC	21	
E4_F	RbP9	GGAATTGGATGAATGGATGTG	21	714
E4_R	RbP10	GCAAAGAAGGCTAAAATGGG	20	
E5_F	RbP11	TTGGGAAAATCTACTTGAACTTTG	24	337
E5_R	RbP12	CACAGGACTTAAATCTATGGGC	22	
E6_F	RbP13	AAATTATGCAATTAAAATGGACTGC	25	348
E6_R	RbP14	CCAAGCAGAGAATGAGGGAG	20	
E7_F	RbP15	ACCATGCTGATAGTGATTGTTG	22	360
E7_R	RbP16	ATGGGCAAAGTCCATGTCTG	20	
E8_F	RbP17	GCAGAGTAGAAGAGGGATGGC	21	558
E8_R	RbP18	TGATTCCAGAGTGAGGGAGC	20	
E9_F	RbP19	TTGACACCTCTAACTTACCCTGC	23	318
E9_R	RbP20	TTTCACCACAATTCTACTTGGC	22	
E10_F	RbP21	TTTATATTGCATGCGAACTCAG	22	469
E10_R	RbP22	GGTAACTGTTATAGGACACACAATTC	26	
E11_F	RbP57	TGATGCATAAAGCACAAATTGT	22	218
E11_R	RbP58	AACGTGAACAAATCTGAAACACTA	24	
E12_F	RbP25	GAGACAAGTGGGAGGCAGTG	20	344
E12_R	RbP26	AAGCAAGAAAAGATTATGGATAACTAC	27	
E13_F	RbP27	TGCTTATGTTCAGTAGTTGTGGTTAC	26	420
E13_R	RbP28	TAGCAGCATACACAGGCAGC	20	
E14_F	RbP29	TAGCAGGCTCTTATTTTTCTTTTTG	25	209
E14_R	RbP30	GATGATCTTGATGCCTTGACCT	22	
E15+16_F	RbP31	CAATGCTGACACAAATAAGG	20	366
E15+16_R	RbP32	AGCATTCCTTCTCCTTAACC	20	
E17_F	RbP33	TCAAAATTGGAAGGCTATTTCC	22	569
E17_R	RbP34	TTAGATGGTTTAGGGTGCTCG	21	
E18_F	RbP35	ATTGTCAATTGGGAATTTCG	20	504
E18_R	RbP36	TGCAAATCCTAGGTGATTCAG	21	
E19_F	RbP37	ATAATCTGTGATTCTTAGCC	20	273
E19_R	RbP38	AAGAAACATGATTTGAACCC	20	
E20_F	RbP39	AAAGAGTGGTAGAAAAGAGG	20	335
E20_R	RbP40	CAGTTAACAAGTAAGTAGGG	20	
E21_F	RbP41	TTTGTTCTTTAAACACACTTTGGG	24	486
E21_R	RbP42	CATAATTACCCTTATCTTTCCAATTC	26	
E22+23_F	RbP43	TCCTTTATAATATGTGCTTCTTACCAG	27	577
E22+23_R	RbP44	TTCTTGGATCAAAATAATCCCC	22	
E24_F	RbP45	TGTCAGTGGTTCTAGGGTAGAGG	23	280
E24_R	RbP46	ATGCCTGGATGAGGTGTTTG	20	
E25_F	RbP47	AAATGAAGTTATTACCTTTGCCTG	24	362
E25_R	RbP48	CTTGGCATGAAAGAAATTGG	20	
E6_Anchor_F	RbP54	AATGCACAAAAAGAAACACC	20	-
E9_Anchor_R	RbP55	GTGATACACAGTAAATTGATC	21	-
E15_Anchor_F	RbP63	CAACTTCTTTTTTTTTTTTAAATTATCTG	30	-
E15_Anchor_R	RbP56	AAGATTATAAAATACTTACTTC	22	-
E16_Anchor_F	RbP60	ATAATCTTTTTTTTTTTCC	19	-
E17_Anchor_F	RbP61	AGTTACTTTTTTTTTTCAT	19	-
E22+23_Anchor_F	RbP62	TTTTTTTTTTTTACTGTTC	19	-

Primers (RbP1–48 and RbP57–58) were used for PCR amplification of promoter and exons 1–25 with flanking intronic sequences. RbP1–48 and RbP57–58 were also used for sequencing of PCR products except amplicons from exons 6, 9, 15, 16, 17, 22 and 23 which were sequenced using a specific primer for antisense and sense stands, listed as follows: RbP14 and RbP54 (exon 6); RbP19 and RbP55 (exon 9); RbP63 and RbP56 (exon 15); RbP60 and RbP32 (exon 16); RbP61 and RbP34 (exons 17); and RbP62 and RbP44 (exon 22 and 23).

Supplementary Table S2. Tumor laterality, tumor focality, and status of germline *RB1* mutation of retinoblastoma patients from two studies

	Previous study[10]			This study		
	n	Positive	Negative	n	Positive	Negative
Bilateral	25	22	3	18	16	2
Unilateral	27	9	18	24	1	23
Total	52	31	21	42	17	25
Multifocal*	30	24	6	21	17	4
Unifocal*	17	3	14	16	0	16
Total*	47	27	20	37	17	20

*Patients with available data on tumor focality. Unavailable data as the impossibility of identifying tumor focality in a large tumor size. There is no overlap of patients between two studies.

Supplementary Table S3. *In silico* pathogenicity analysis of novel *RB1* variants

**Cases**	**Mutation types**	**Prediction of pathogenicity**			
		**MutPred2**	**MutationTaster**	**Human Splicing Finder**	**MaxEntScan**
321 N94	Frame shift	Disease-associated	Disease causing	-	-
133 N118	Frame shift	Disease-associated	Disease causing	-	-
196 N138	Splice	Disease-associated	Disease causing	Broken WT donor site	Broken WT donor site

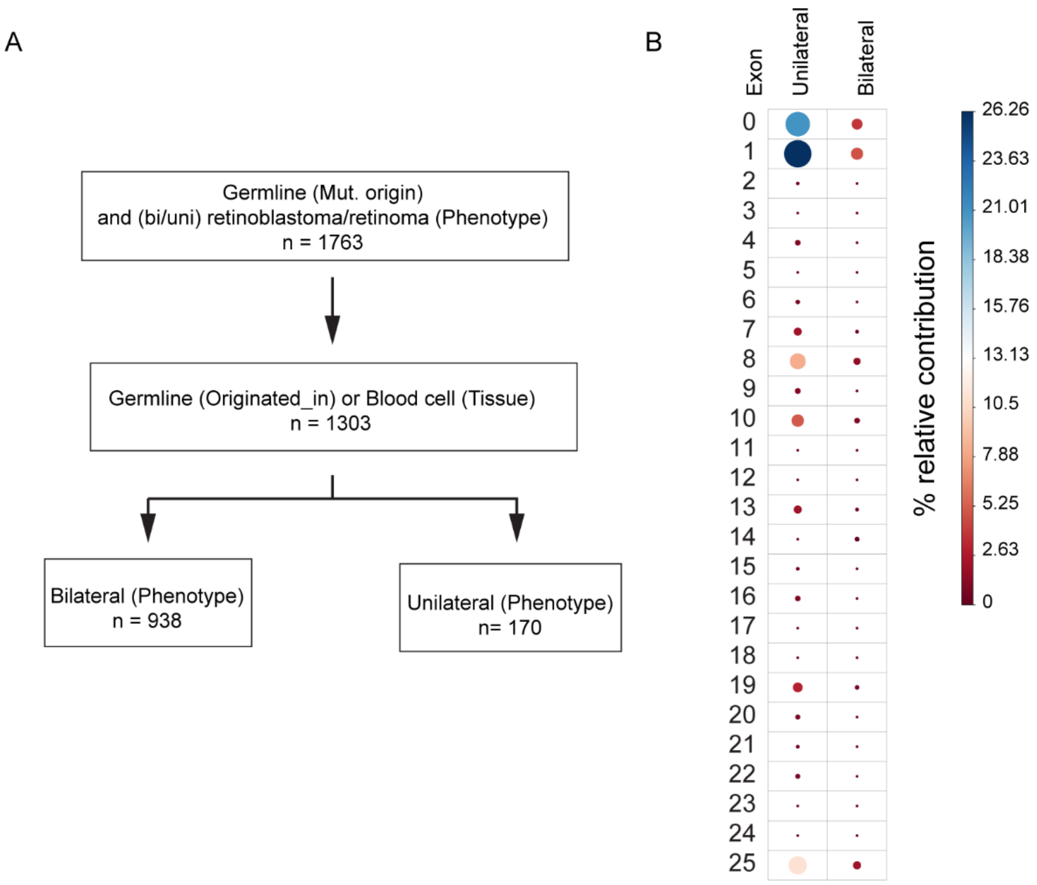

Supplementary Figure S1. Data extracted from rb1-lsdb database. (**A**) Criteria for selection of data on germline RB1 mutations from rb1-lsdb database. n = number of mutations in promoter and exons 1–25 of the *RB1* gene. (**B**) The relative contribution (%) of each cell to the total Chi-squared score indicates the degree of dependency between exons and tumor laterality of the contingency table (p-value = 1.075×10^{-8}).
